# Two Cu^II^ complexes of 3,4,5-tri­methyl-1*H*-pyrazole

**DOI:** 10.1107/S2056989018002359

**Published:** 2018-02-16

**Authors:** Collin J. Vincent, Ian D. Giles, Jeffrey R. Deschamps

**Affiliations:** a Department of Chemistry, Washington College, 300 Washington Ave., Chestertown, MD 21620, USA; b CBMSE, Code 6910, U. S. Naval Research Laboratory, 4555 Overlook Ave, SW, Washington, DC 20375, USA

**Keywords:** crystal structure, tri­methyl­pyrazole, copper(II), complex, metal organic, nitrate, chloride

## Abstract

The crystal structure of complexes of 3,4,5-tri­methyl-1*H*-pyrazole with CuCl_2_·2H_2_O and Cu(NO_3_)_2_·2.5H_2_O are presented, namely di-μ-chlorido-bis[chloridobis(3,4,5-trimethyl-1*H*-pyrazole-κ*N* ^2^)copper(II)], [Cu_2_Cl_4_(C_6_H_10_N_2_)_4_] (**1**) and aquatetrakis(3,4,5-trimethyl-1*H*-pyrazole-κ*N* ^2^)copper(II) dinitrate, [Cu(C_6_H_10_N_2_)_4_(H_2_O)](NO_3_)_2_ (**2**), and compared to the previously determined structures for 3-methyl-1*H*-pyrazole and 3,5-di­methyl-1*H*-pyrazole. CuCl_2_ forms a 2:1 ligand-to-metal chloride-bridged complex with 3,4,5-tri­methyl-1*H*-pyrazole, with a square-pyramidal coordination geometry about each copper(II) center. Similarly to the previously obtained 3,5-di­methyl-1*H*-pyrazole complex with CuCl_2_, the pyrazole ligands are *cis* to each other, with two chloride ions bridging the two copper(II) centers, and a terminal chloride ion occupying the axial position. Cu(NO_3_)_2_ forms a 4:1 ligand-to-metal complex with 3,4,5-tri­methyl-1*H*-pyrazole that is also arranged in a square-pyramidal geometry about Cu^II^. The newly obtained copper(II) complex has the same coordination geometry as the 3,5-di­methyl-1*H*-pyrazole complex, including an axial water mol­ecule, two nitrate ions hydrogen-bonded to the water mol­ecule, and four pyrazole ligands in the equatorial plane, suggesting that similar steric forces are at play in the formation of these complexes.

## Abstract 2

The crystal structures of two Cu^II^ complexes of 1-*H*-3,4,5-tri­methyl­pyrazole, *cis*-[{CuCl[3,4,5-(CH_3_)_3_(C_3_H_2_N_2_)]_2_}_2_(μ-Cl)_2_] and [Cu{3,4,5-(CH_3_)_3_(C_3_HN_2_)}_4_(H_2_O)](NO_3_)_2_, are presented and compared to the 3-methyl-1*H*-pyrazole and 3,5-di­methyl-1*H*-pyrozole analogues.

## Chemical context   

Pyrazoles are a useful class of mol­ecules because they coordinate with metal ions, form conjugated π-systems, and can be tuned electronically and sterically through a number of possible substituent groups. It is therefore important to gain a better understanding of how changes in reaction conditions, including solvent, substituents, and counter-ions, affect the structures of compounds incorporating pyrazole and its deriv­atives. Previous work using mono- and dimethyl pyrazole ligands demonstrated the effect of the counter-ion on the final structure and electronic properties of their respective Cu­^II^ complexes from water (Giles *et al.*, 2015 ▸). Absent in this analysis were complexes incorporating 1-*H*-3,4,5-tri­methyl­pyrazole. Work presented herein adds structural determinations of complexes of 1-*H*-3,4,5-tri­methyl­pyrazole under the same reaction conditions to complete the series. Complexes incorporating this final ligand are important to obtain a complete understanding of how different pyrazole substituents and their locations affect the coordination environment about the central Cu^II^ atom. CuCl_2_ and Cu(NO_3_)_2_ were used to assess counter-ion effects on the crystal structure in a manner consistent with the previous work.
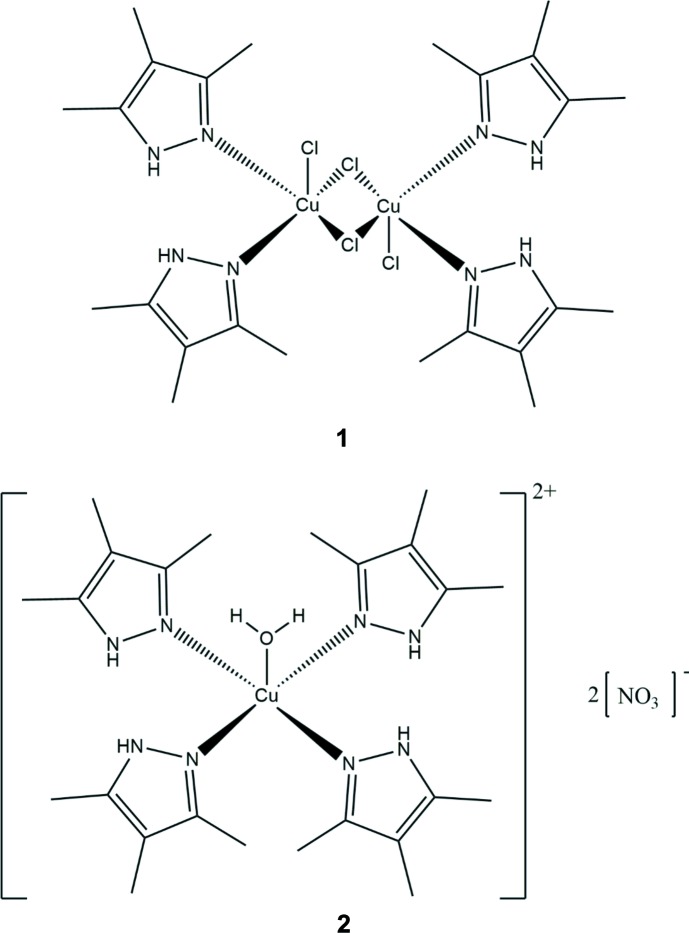


## Structural commentary   

**Figure 1:**
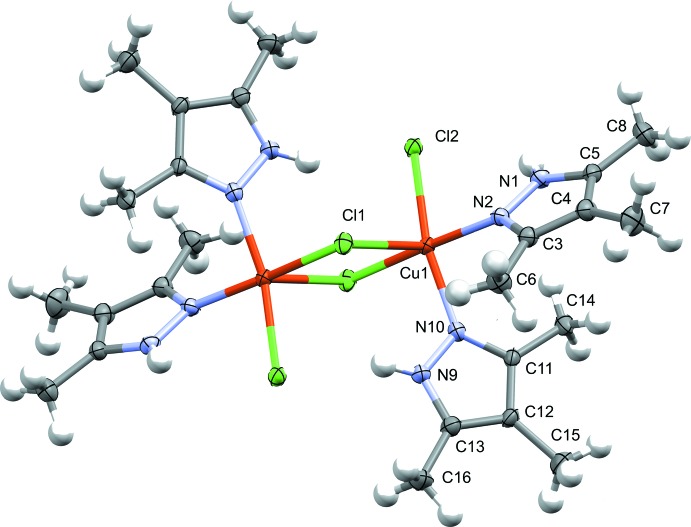
Mol­ecular structure of **1**, showing the atom-labeling scheme. Displacement ellipsoids are drawn at the 50% probability level. The complex resides on an inversion center, with half of the mol­ecule generated by the symmetry operator −*x*, 1 − *y*, 1 − *z*. Only one set of disordered methyl protons are shown. Hydrogen-atom labels are omitted for clarity.

In the CuCl_2_ complex with 1-*H*-3,4,5-tri­methyl­pyrazole (**1**, Fig. 1 ▸), there are two tri­methyl­pyrazole ligands and three chloride ions bound to each Cu^II^ center. Two of the chloride ions bridge asymmetrically to a second copper(II), which is related to the first Cu^II^ by an inversion center. The overall geometry around each Cu^II^ center is square pyramidal, with the axial position occupied by the elongated bridging chloride contact, and the equatorial positions occupied by the two tri­methyl­pyrazole ligands in a *cis* configuration, one terminal chloride ion, and the shorter bridging chloride contact. In **1**, the tri­methyl­pyrazole ligands are tilted off-perpendicular from the basal plane of the square-pyramidal Cu^II^ coordination environment. The dihedral angles of the pyrazole ligands to the basal plane and to each other are as follows: between the mean N9/N10/C11–C13 plane and the mean Cl2/Cl1/N2/N10 plane, 53.9 (2)°; between the mean N2/N1/C3–C5 plane and the mean Cl2/Cl1/N2/N10 plane, 47.1 (2)°; between the mean N9/N10/C11–C13 and the mean N2/N1/C3–C5 plane, 51.5 (2)°. The tri­methyl­pyrazole ligand is not deprotonated in the complex as there are two chloride ions per Cu^II^ ion. Additionally, the bond distances within the tri­methyl­pyrazole ring are more characteristic of a non-aromatic, conjugated ring [C3—C4, C11—C12, 1.403 (6) and 1.410 (6) Å; C4—C5, C12—C13, 1.383 (6)–1.388 (6) Å; C13—N9, C11—N10, 1.341 (5) Å, C3—N2, C5—N1, 1.335 (5) and 1.339 (5) Å; N9—N10, N1—N2, 1.353 (5) and 1.356 (5) Å], rather than the heteroaromatic species obtained upon deprotonation (Allen *et al.*, 1987 ▸). The structure produced is similar to the previously reported 1-*H*-3,5-di­methyl­pyrazole Cu^II^ complex. These structures differ from the 1-*H*-3-methyl­pyrazole-Cu^II^ complex primarily through the positioning of the pyrazole ligands, which are oriented *trans* to one another in the mono­methyl­pyrazole complex rather than *cis* as in the di- and tri­methyl­pyrazole complexes (Giles *et al.*, 2015 ▸).

**Figure 2:**
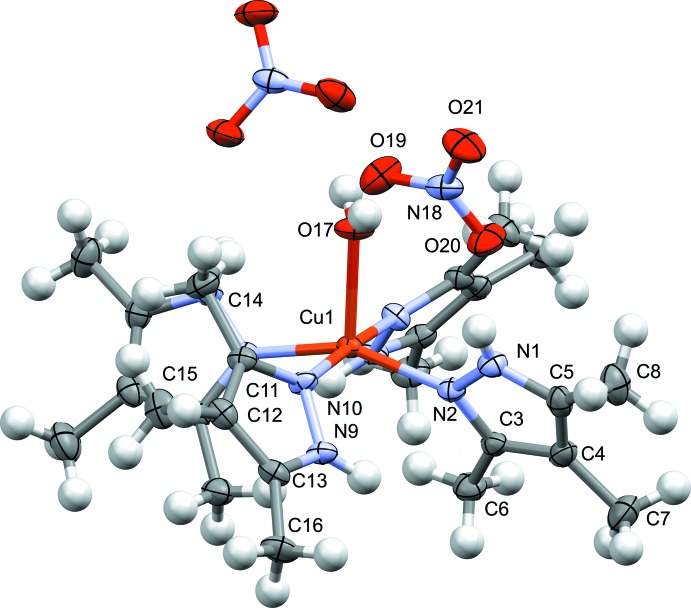
Mol­ecular structure of **2**, showing the atom-labeling scheme. Displacement ellipsoids are drawn at the 50% probability level. Only one set of disordered methyl protons are shown. The Cu—OH_2_ bond resides on a twofold rotation axis, with half of the mol­ecule generated by the symmetry operator 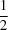 − *x*, *y*, 1 − *z*. Hydrogen-atom labels are omitted for clarity.

In **2** (Fig. 2 ▸), the complex produced by mixing Cu(NO_3_)_2_ and 1-*H*-3,4,5-tri­methyl­pyrazole, the structure consists of a single Cu^II^ center exhibiting a square-pyramidal coordination geometry, oriented such that the pyrazole ligands occupy the four planar positions around Cu^II^, with a water mol­ecule occupying the axial position. The pyrazole ligands are oriented so that the non-coordinated pyrazole nitro­gen atoms are *cis* across the N—Cu—N bonds on opposite sides of the structure, and are *trans* across the N—Cu—N bonds on adjacent pyrazoles. Additionally, in **2** as in **1**, the tri­methyl­pyrazole ligands are tilted off-perpendicular from the basal plane of the square-pyramidal Cu^II^ coordination environment. The dihedral angles of the pyrazole ligands to the basal plane and to each other are as follows: between the mean N9/N10/C11–C13 and the mean N2/N10/N10*/N2* plane, 49.2 (2)°; between the mean N2/N1/C3–C5 plane and the mean N2/N10/N10*/N2* plane, 61.3 (2)°; between the mean N9/N10/C11—C13 and the mean N2/N1/C3–C5 plane, 78.7 (2)° (N10* and N2* are symmetry-equivalent atoms generated by the symmetry operator 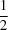 − *x*, *y*, 1 − *z*). The nitrate ions are not directly coordinated to Cu^II^. This structure is similar to that obtained from 1-*H*-3,5-di­methyl­pyrazole and Cu(NO_3_)_2_ in water, which reinforces the conclusion that steric effects likely play a part in determining how the ligands orient themselves in this complex (Giles *et al.*, 2015 ▸). As in **1**, the bond distances within the pyrazole ring are indicative of discrete single and double bonds [C3—C4, C11—C12, 1.401 (4)–1.405 (4) Å; C4—C5, C12—C13, 1.379 (4) and 1.373 (4) Å, respectively; C13—N9, 1.341 (3) Å; C11—N10, 1.336 (3) Å, C3—N2, 1.332 (3) Å; C5—N1, 1.349 (4) Å; N9—N10, 1.369 (3) Å; N1—N2, 1.358 (3) Å] providing further evidence for a neutral tri­methyl­pyrazole ligand (Allen *et al.*, 1987 ▸).

## Supra­molecular features   

**Figure 3:**
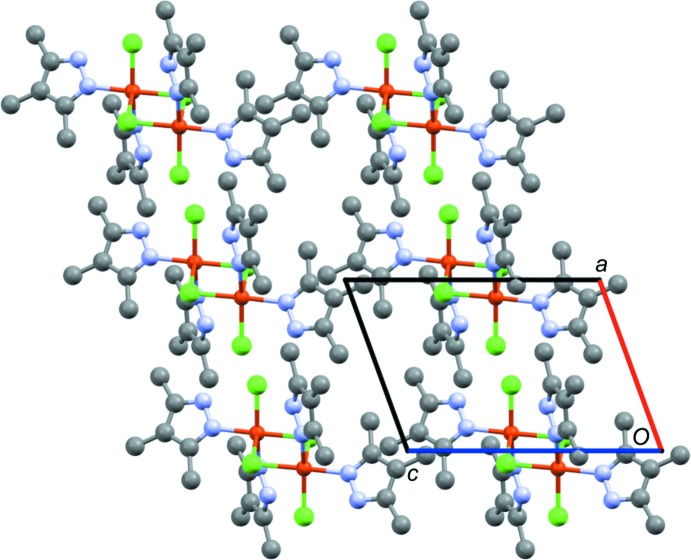
Packing of **1** viewed down the crystallographic *b*-axis direction, highlighting the alignment of the copper complexes in the same orientation throughout the crystal. Hydrogen atoms are omitted for clarity.

In **1**, there is limited intra­molecular hydrogen bonding, specifically between the terminal chloride ions (Cl2) and very weak intermolecular interactions involving the same Cl2 atoms and N—H groups of adjacent complexes (Table 1 ▸). The distances between translation-related Cu atoms of adjacent complexes is 8.89 (2) Å, which is greater than the 8.68 (2) Å for the comparable Cu⋯Cu distance in the di­methyl­pyrazole complex, suggesting additional steric crowding due to the third methyl group. The packing (Fig. 3 ▸) is also different in that the tri­methyl­pyrazole complexes are oriented in the same direction within the crystal, whereas the di­methyl­pyrazole complexes alternate their orientation. Both the di- and tri­methyl­pyrazole complexes pack in space group *P* 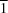.

**Figure 4:**
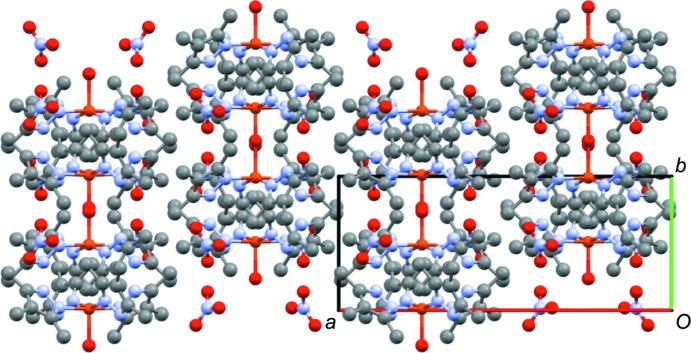
Packing of **2** viewed down the crystallographic *c*-axis direction, highlighting the alternating columns of stacked copper complexes. Hydrogen atoms are omitted for clarity.

In **2**, there is hydrogen bonding present between the oxygen atoms of the nitrate ions and both the pyrazole N—H and coordinated water O—H atoms on the complex, limiting the positional disorder of the nitrate ions (Table 2 ▸). Surprisingly, **2** packs more closely together (Fig. 4 ▸) than its congener incorporating 1-*H*-3,5-di­methyl­pyrazole (Giles *et al.*, 2015 ▸). The positioning of the pyrazole ligands in the tri­methyl­pyrazole complex is such that the pyrazole–pyrazole overlap occurs between two different portions of the pyrazole ring, allowing for a closer contact [centroid–centroid distance between N9/N10/C11–C13 rings = 4.49 (2) Å, distance between ring planes = 3.35 (2) Å], likely the result of pyrazole ring polarization that leaves one region electron-withdrawn while the other is more electron-rich. In the di­methyl­pyrazole complex, the pyrazole ligands overlap with the same region of the ring, which have similar electronic properties and therefore are more repulsive, increasing the ring–ring overlap distance as measured between the comparable ring centroids [4.98 (2) Å] and the inter­plane distance [3.97 (2) Å]. The result is closer packing for the tri­methyl­pyrazole complex [10.06 (2) Å between Cu^II^ centers of complexes in adjacent columns, 7.89 (2) Å between Cu^II^ centers of complexes within stacked columns] when compared to the di­methyl­pyrazole [10.15 (2) Å between Cu^II^ centers in adjacent complexes, 8.23 (2) Å between Cu^II^ centers in stacked complexes]. Both structures crystallize in space group *I*2/*a* (reported as *C*2/*c* for the di­methyl­pyrazole complex).

## Database survey   

A search of the CSD (Groom *et al.*, 2016 ▸; Version 5.38, May 2017 update) for structures containing 1-*H*-3,4,5-tri­methyl­pyrazole yields only 17 entries. The structure with CSD refcode FITQEE is of the neutral ligand only (Infantes *et al.*, 1999 ▸). In this structure, one tri­methyl­pyrazole mol­ecule resides on a twofold rotation axis, with positional disorder of the pyrazole N—H proton, and a C—C bond length of 1.389 Å, a C—N bond length of 1.341 Å, and a N—N bond length of 1.346 Å. The C—C and C—N bond lengths are equivalent because of the twofold rotation. The other tri­methyl­pyrazole mol­ecule does not reside on a symmetry element, but still contains C—C and C—N bonds that are close in distance (C—C range from 1.385 to 1.388 Å and C—N is 1.336 Å), with an N—N distance of 1.357 Å. These distances are comparable to those seen in **1** and **2**, although both **1** and **2** show wider ranges of bond lengths than the free ligand.

The closest match to **1**, CSD refcode CENJIO, is a fluoride-bridged Cu^II^ complex containing three 1-*H*-3,4,5,-tri­methyl­pyrazole ligands per copper(II) center (Rietmeijer *et al.*, 1984 ▸) and tetra­fluoro­borate as counter-ion. In this complex, the pyrazole C—C (1.356–1.396 Å), C—N (1.321–1.334 Å), and N—N (1.355–1.356 Å) bond lengths are as expected in a neutral pyrazole group, and similar to those seen in **1** and **2**, athough the bond lengths in CENJIO span a wider range. The closest match to **2**, CSD refcode RIDHAP, is a 4: 1 1-*H*-3,4,5-tri­methyl­pyrazole complex with coordinated perchlorate anions (Ardizzoia *et al.*, 2013 ▸). The pyrazole C—C (1.363–1.411 Å), C—N (1.329–1.356 Å), and N—N (1.352–1.353 Å) bond lengths are within expected lengths for a neutral pyrazole group and are comparable to, but cover a wider range of distances than, those in **1** and **2**.

A complex structurally similar to **1** is found in the CSD with refcode NURPEX (Giles *et al.*, 2015 ▸), incorporating 1-*H*-3,5-di­methyl­pyrazole. The C—C (1.345–1.417 Å), C—N (1.308–1.365 Å), and N—N (1.338–1.374 Å) bond distances within the pyrazole group are similar to those in **1**, but cover a wider range. Complexes structurally similar to **2** can be found in the CSD with refcodes FAYTOO (Pervukhina *et al.*, 1986 ▸), MIFYUW (Denisova *et al.*, 2006 ▸), and YUXSEP/YUXSEP01 (Pervukhina *et al.*, 1995 ▸ and Giles *et al.*, 2015 ▸, respectively), all incorporating 1-*H*-3,5-di­methyl­pyrazole as the ligand with perchlorate, tri­fluoro­methyl­sulfonate, and nitrate anions, respectively. In these complexes, the C—C, C—N, and N—N bond lengths are as expected for neutral pyrazole ligands, with C—C bond-length ranges of 1.263–1.520 Å (FAYTOO), 1.366–1.389 Å (MIFYUW), and 1.369–1.400 Å (YUXSEP01); C—N bond length ranges of 1.270–1.433 Å (FAYTOO), 1.329–1.346 Å (MIFYUW), and 1.334–1.350 Å (YUXSEP01); and N—N bond length ranges of 1.322–1.477 Å (FAYTOO), 1.361–1.375 Å (MIFYUW), and 1.360–1.365 Å (YUXSEP01).

## Synthesis and crystallization   

All manipulations were carried out in air at room temperature with reagents as obtained from the manufacturer, unless otherwise stated. 3-Methyl-2,4-penta­nedione was purchased from Alfa–Aesar, while the hydrazine monohydrate was purchased from Sigma–Aldrich. CuCl_2_·2H­_2_O was purchased from Aldrich, and Cu(NO_3_)_2_·2.5H­_2_O was purchased from Fisher. Deionized water was used in all reactions.

**1**-***H*** **-3,4,5-tri­methyl­pyrazole:** Following literature procedure (Morin *et al.*, 2011 ▸), clear, colorless hydrazine monohydrate (5.08 mL, 105 mmol) was slowly dissolved in 20 mL of methanol. Yellow 3-methyl-2,4-penta­nedione (11.9 g, 104 mmol) was dissolved in 50 mL of methanol and cooled in an ice bath. The hydrazine monohydrate in methanol was added dropwise to the stirring 3-methyl-2,4-penta­nedione solution. The reaction was stirred for about 15 minutes, during which time condensation appeared on the inside of the flask. The reaction remained clear yellow. The reaction mixture was then refluxed for about an hour. The methanol was evaporated using rotary evaporation at 333K, resulting in an off-white solid. The product was recrystallized from hot hexa­nes, producing pale-yellow crystals, collected by vacuum filtration (9.47 g, 82.5%). Identity confirmed by ^1^H NMR and IR spectroscopy.

***cis*** **-[{CuCl[3,4,5-(CH_3_)_3_(C_3_H_2_N_2_)]_2_}_2_(μ-Cl)_2_] (1):** 1-*H*-3,4,5-tri­methyl­pyrazole (0.16704 g, 1.5163 mmol) was dissolved in 5 mL of H_2_O, with 1 mL of acetone added to aid dissolution. A light-blue solution of 0.13531 g (0.79369 mmol) CuCl_2_·2H_2_O in 5 mL H_2_O was added *via* pipette to this solution while stirring. A 1 mL rinse of the Cu^II^ vessel with H_2_O was added to the reaction. There was an immediate change of color to light green as the Cu^II^ solution was added, which darkened upon further addition of the Cu^II^ solution, reaching dark green. Upon complete addition of Cu^II^, the solution became teal green with a small amount of precipitate. The reaction was stirred overnight, filtered, and the solvent slowly evaporated to yield dark-green crystals.

**[Cu{3,4,5-(CH_3_)_3_(C_3_HN_2_)}_4_(H_2_O)](NO_3_)_2_ (2)** 1-H-3,4,5-tri­methyl­pyrazole (0.16601 g, 1.5070 mmol) was dissolved in 5 mL of H_2_O, with 1 mL of acetone added to aid dissolution. A light-blue solution of 0.18296 g (0.78655 mmol) Cu(NO_3_)_2_·2.5 H_2_O in 5 mL H_2_O was added via pipette to this solution while stirring. A 1 mL rinse of the Cu^II^ vessel with H_2_O was added to the reaction. There was an immediate change of color to light green as the Cu^II^ solution was added, which darkened upon further addition of the Cu^II^ solution, reaching dark green. Upon complete addition of Cu^II^, the solution became teal green with a small amount of precipitate. The reaction was stirred overnight, filtered, and the solvent slowly evaporated to yield dark-blue crystals.

## Refinement   

Crystal data, data collection and structure refinement details are summarized in Table 3 ▸. For **1**, five reflections (110), (100), (010), (001), and (111) were omitted from the final refinement on account of beamstop truncation. For **2**, five reflections (110), (200), (011), (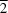
02), and (002) were omitted from the final refinement on account of beamstop truncation. N—H H atoms were freely refined. Hydrogen atoms on methyl groups in both **1** and **2** were placed at calculated positions incorporating two-position rotational disorder and refined using a riding model, C—H = 0.98 Å and *U*_iso_(H) = 1.5*U*_eq_(C).

## Supplementary Material

Crystal structure: contains datablock(s) global, 1, 2. DOI: 10.1107/S2056989018002359/vm2208sup1.cif

Structure factors: contains datablock(s) 1. DOI: 10.1107/S2056989018002359/vm22081sup2.hkl

Supporting information file. DOI: 10.1107/S2056989018002359/vm22081sup4.cdx

Structure factors: contains datablock(s) 2. DOI: 10.1107/S2056989018002359/vm22082sup3.hkl

Supporting information file. DOI: 10.1107/S2056989018002359/vm22082sup5.cdx

CCDC references: 1823018, 1823017

Additional supporting information:  crystallographic information; 3D view; checkCIF report

## Figures and Tables

**Table 1:** Hydrogen-bond geometry (Å, °) for **1** [Chem scheme1]

*D*—H⋯*A*	*D*—H	H⋯*A*	*D*⋯*A*	*D*—H⋯*A*
C14—H14*E*⋯N2	0.98	2.49	3.199 (5)	129
C14—H14*E*⋯N1	0.98	2.54	3.401 (6)	147
C14—H14*E*⋯N2	0.98	2.49	3.199 (5)	129
C14—H14*E*⋯N1	0.98	2.54	3.401 (6)	147
C16—H16*F*⋯Cl2^i^	0.98	2.85	3.776 (5)	158
C6—H6*E*⋯N9	0.98	2.67	3.521 (6)	146
C6—H6*E*⋯N10	0.98	2.55	3.240 (6)	128
C6—H6*B*⋯Cl1^ii^	0.98	2.86	3.692 (5)	144
N1—H1⋯Cl2	0.75 (4)	2.66 (4)	3.102 (4)	120 (3)
N1—H1⋯Cl2^iii^	0.75 (4)	2.54 (4)	3.214 (4)	151 (3)
N9—H9⋯Cl1^ii^	0.86 (6)	2.94 (6)	3.506 (4)	125 (5)
N9—H9⋯Cl2^ii^	0.86 (6)	2.37 (6)	3.188 (4)	159 (5)

Symmetry codes: (i) 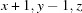; (ii) 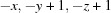; (iii) 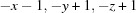.

**Table 2:** Hydrogen-bond geometry (Å, °) for **2** [Chem scheme1]

*D*—H⋯*A*	*D*—H	H⋯*A*	*D*⋯*A*	*D*—H⋯*A*
C6—H6*E*⋯N9^i^	0.98	2.50	3.355 (4)	145
C6—H6*E*⋯N10^i^	0.98	2.60	3.274 (4)	126
C6—H6*F*⋯O19^ii^	0.98	2.56	3.367 (4)	140
C6—H6*E*⋯N9^i^	0.98	2.50	3.355 (4)	145
C6—H6*E*⋯N10^i^	0.98	2.60	3.274 (4)	126
C14—H14*B*⋯O17	0.98	2.38	3.193 (3)	140
C16—H16*B*⋯O20^iii^	0.98	2.62	3.525 (4)	153
C16—H16*B*⋯N18^iii^	0.98	2.66	3.342 (4)	127
C16—H16*D*⋯O21^ii^	0.98	2.57	3.430 (4)	146
C8—H8*D*⋯O20	0.98	2.60	3.411 (4)	140
N1—H1⋯O20	0.75 (3)	2.10 (3)	2.801 (3)	158 (3)
N9—H9⋯N2	0.92 (3)	2.54 (3)	3.039 (3)	114 (2)
N9—H9⋯O21^ii^	0.92 (3)	2.24 (3)	3.033 (3)	144 (2)
N9—H9⋯O19^ii^	0.92 (3)	2.52 (3)	3.150 (3)	126 (2)
O17—H17⋯O19	0.79 (3)	1.97 (3)	2.755 (3)	174 (3)
C6—H6*F*⋯O19^ii^	0.98	2.56	3.367 (4)	140
C6—H6*E*⋯N9^i^	0.98	2.50	3.355 (4)	145
C6—H6*E*⋯N10^i^	0.98	2.60	3.274 (4)	126
C6—H6*E*⋯N9^i^	0.98	2.50	3.355 (4)	145
C6—H6*E*⋯N10^i^	0.98	2.60	3.274 (4)	126
C14—H14*B*⋯O17	0.98	2.38	3.193 (3)	140
C16—H16*B*⋯O20^iii^	0.98	2.62	3.525 (4)	153
C16—H16*B*⋯N18^iii^	0.98	2.66	3.342 (4)	127
C16—H16*D*⋯O21^ii^	0.98	2.57	3.430 (4)	146
C8—H8*D*⋯O20	0.98	2.60	3.411 (4)	140
N1—H1⋯O20	0.75 (3)	2.10 (3)	2.801 (3)	158 (3)
N9—H9⋯N2	0.92 (3)	2.54 (3)	3.039 (3)	114 (2)
N9—H9⋯O21^ii^	0.92 (3)	2.24 (3)	3.033 (3)	144 (2)
N9—H9⋯O19^ii^	0.92 (3)	2.52 (3)	3.150 (3)	126 (2)
O17—H17⋯O19	0.79 (3)	1.97 (3)	2.755 (3)	174 (3)

Symmetry codes: (i) 
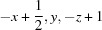
; (ii) 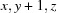; (iii) 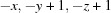.

**Table 3:** Experimental details

	**1**	**2**
Crystal data		
Chemical formula	[Cu_2_(C_24_H_40_Cl_4_N_8_)]	[Cu(C_6_H_10_N_2_)_4_(H_2_O)](NO_3_)_2_
*M*_r_	709.52	646.21
Crystal system, space group	Triclinic, *P* 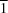	Monoclinic, *I*2/*a*
Temperature (K)	150	150
*a*, *b*, *c* (Å)	8.887 (3), 9.460 (3), 11.214 (3)	20.107 (7), 7.8939 (16), 20.472 (4)
α, β, γ (°)	85.408 (4), 69.978 (4), 64.097 (4)	90, 102.651 (2), 90
*V* (Å^3^)	794.1 (4)	3170.5 (14)
*Z*	1	4
Radiation type	Mo *K*α	Mo *K*α
μ (mm^−1^)	1.70	0.75
Crystal size (mm)	0.07 × 0.05 × 0.03	0.16 × 0.10 × 0.03

Data collection		
Diffractometer	Bruker SMART APEXII CCD	Bruker SMART APEXII CCD
Absorption correction	Multi-scan (*SADABS*; Bruker, 2014 ▸)	Multi-scan (*SADABS*; Bruker, 2014 ▸)
*T*_min_, *T*_max_	0.660, 0.746	0.654, 0.746
No. of measured, independent and observed [*I* > 2σ(*I*)] reflections	7770, 3620, 2472	13530, 3636, 2719
*R*_int_	0.048	0.057
(sin θ/λ)_max_ (Å^−1^)	0.652	0.650

Refinement		
*R*[*F* ^2^ > 2σ(*F* ^2^)], *wR*(*F* ^2^), *S*	0.046, 0.138, 0.88	0.045, 0.135, 0.91
No. of reflections	3620	3636
No. of parameters	180	203
H-atom treatment	H atoms treated by a mixture of independent and constrained refinement	H atoms treated by a mixture of independent and constrained refinement
Δρ_max_, Δρ_min_ (e Å^−3^)	0.87, −0.62	0.78, −0.61

Computer programs: *APEX2*, *SAINT* and *XPREP* (Bruker, 2014 ▸), *SHELXL2016/6* (Sheldrick, 2015 ▸), *SHELXTL* (Sheldrick, 2008 ▸) and *X-SEED* (Barbour, 2001 ▸).

## supplementary crystallographic information

### Di-µ-chlorido-bis[chloridobis(3,4,5-trimethyl-1H-pyrazole-\ κN2)copper(II)] (1) Crystal data

**Table:**

[Cu_2_Cl_4_(C_6_H_10_N_2_)_4_]	*Z* = 1
*M**_r_* = 709.52	*F*(000) = 366
Triclinic, *P*1	*D*_x_ = 1.484 Mg m^−^^3^
*a* = 8.887 (3) Å	Mo *K*α radiation, λ = 0.71073 Å
*b* = 9.460 (3) Å	Cell parameters from 1932 reflections
*c* = 11.214 (3) Å	θ = 5.8–55.1°
α = 85.408 (4)°	µ = 1.70 mm^−^^1^
β = 69.978 (4)°	*T* = 150 K
γ = 64.097 (4)°	Block, green
*V* = 794.1 (4) Å^3^	0.07 × 0.05 × 0.03 mm

### Di-µ-chlorido-bis[chloridobis(3,4,5-trimethyl-1H-pyrazole-\ κN2)copper(II)] (1) Data collection

**Table:**

Bruker SMART APEXII CCD diffractometer	3620 independent reflections
Radiation source: fine focus sealed tube	2472 reflections with *I* > 2σ(*I*)
Graphite monochromator	*R*_int_ = 0.048
ω scans	θ_max_ = 27.6°, θ_min_ = 3.8°
Absorption correction: multi-scan (SADABS; Bruker, 2014)	*h* = −11→11
*T*_min_ = 0.660, *T*_max_ = 0.746	*k* = −12→12
7770 measured reflections	*l* = −14→14

### Di-µ-chlorido-bis[chloridobis(3,4,5-trimethyl-1H-pyrazole-\ κN2)copper(II)] (1) Refinement

**Table:**

Refinement on *F*^2^	0 restraints
Least-squares matrix: full	Hydrogen site location: mixed
*R*[*F*^2^ > 2σ(*F*^2^)] = 0.046	H atoms treated by a mixture of independent and constrained refinement
*wR*(*F*^2^) = 0.138	*w* = 1/[σ^2^(*F*_o_^2^) + (0.094*P*)^2^] where *P* = (*F*_o_^2^ + 2*F*_c_^2^)/3
*S* = 0.88	(Δ/σ)_max_ < 0.001
3620 reflections	Δρ_max_ = 0.87 e Å^−^^3^
180 parameters	Δρ_min_ = −0.62 e Å^−^^3^

### Di-µ-chlorido-bis[chloridobis(3,4,5-trimethyl-1H-pyrazole-\ κN2)copper(II)] (1) Special details

**Table:**

Geometry. All esds (except the esd in the dihedral angle between two l.s. planes) are estimated using the full covariance matrix. The cell esds are taken into account individually in the estimation of esds in distances, angles and torsion angles; correlations between esds in cell parameters are only used when they are defined by crystal symmetry. An approximate (isotropic) treatment of cell esds is used for estimating esds involving l.s. planes.
Refinement. Reflections omitted from final refinement on account of beamstop truncation: (h k l) 1 1 0; 1 0 0; 0 1 0; 0 0 1; 1 1 1

### Di-µ-chlorido-bis[chloridobis(3,4,5-trimethyl-1H-pyrazole-\ κN2)copper(II)] (1) Fractional atomic coordinates and isotropic or equivalent isotropic displacement parameters (Å^2^)

**Table:**

	*x*	*y*	*z*	*U*_iso_*/*U*_eq_	Occ. (<1)
Cu1	−0.10296 (6)	0.41056 (5)	0.43296 (5)	0.01719 (16)	
Cl1	−0.07621 (13)	0.41784 (11)	0.63096 (9)	0.0204 (2)	
Cl2	−0.38433 (13)	0.61388 (11)	0.50173 (10)	0.0216 (2)	
N9	0.2606 (5)	0.1725 (4)	0.3862 (3)	0.0218 (8)	
N2	−0.1329 (4)	0.3931 (4)	0.2643 (3)	0.0197 (7)	
N10	0.0972 (4)	0.1950 (4)	0.3908 (3)	0.0201 (7)	
N1	−0.2889 (5)	0.3999 (4)	0.2661 (4)	0.0201 (7)	
C11	0.1218 (5)	0.0584 (5)	0.3422 (4)	0.0186 (8)	
C13	0.3867 (6)	0.0275 (5)	0.3368 (4)	0.0222 (9)	
C3	−0.0190 (6)	0.3394 (5)	0.1455 (4)	0.0211 (9)	
C5	−0.2781 (6)	0.3542 (5)	0.1527 (4)	0.0224 (9)	
C4	−0.1062 (6)	0.3149 (5)	0.0708 (4)	0.0252 (9)	
C14	−0.0313 (6)	0.0362 (5)	0.3344 (4)	0.0250 (9)	
H14D	0.0120	−0.0703	0.2965	0.037*	0.5
H14F	−0.1182	0.0502	0.4201	0.037*	0.5
H14E	−0.0880	0.1141	0.2814	0.037*	0.5
H14B	−0.1415	0.1330	0.3688	0.037*	0.5
H14A	−0.0113	0.0125	0.2453	0.037*	0.5
H14C	−0.0415	−0.0514	0.3840	0.037*	0.5
C12	0.3018 (6)	−0.0507 (5)	0.3073 (4)	0.0213 (9)	
C16	0.5772 (6)	−0.0243 (5)	0.3215 (5)	0.0316 (11)	
H16B	0.6475	−0.1334	0.2839	0.047*	0.5
H16A	0.6221	0.0442	0.2656	0.047*	0.5
H16C	0.5873	−0.0176	0.4050	0.047*	0.5
H16D	0.5904	0.0622	0.3525	0.047*	0.5
H16F	0.6158	−0.1154	0.3707	0.047*	0.5
H16E	0.6506	−0.0536	0.2313	0.047*	0.5
C6	0.1712 (6)	0.3110 (6)	0.1056 (4)	0.0282 (10)	
H6D	0.2313	0.2721	0.0155	0.042*	0.5
H6F	0.1766	0.4099	0.1180	0.042*	0.5
H6E	0.2305	0.2325	0.1571	0.042*	0.5
H6B	0.1942	0.3375	0.1782	0.042*	0.5
H6A	0.2490	0.1997	0.0757	0.042*	0.5
H6C	0.1951	0.3771	0.0366	0.042*	0.5
C8	−0.4341 (6)	0.3511 (6)	0.1316 (5)	0.0308 (10)	
H8B	−0.4000	0.3145	0.0428	0.046*	0.5
H8A	−0.4720	0.2794	0.1881	0.046*	0.5
H8C	−0.5325	0.4575	0.1500	0.046*	0.5
H8D	−0.5363	0.3864	0.2111	0.046*	0.5
H8F	−0.4643	0.4215	0.0658	0.046*	0.5
H8E	−0.4038	0.2434	0.1040	0.046*	0.5
C15	0.3862 (7)	−0.2166 (5)	0.2485 (5)	0.0344 (11)	
H15B	0.2947	−0.2410	0.2383	0.052*	0.5
H15A	0.4762	−0.2268	0.1650	0.052*	0.5
H15C	0.4428	−0.2900	0.3038	0.052*	0.5
H15D	0.5144	−0.2642	0.2331	0.052*	0.5
H15F	0.3329	−0.2785	0.3065	0.052*	0.5
H15E	0.3663	−0.2152	0.1676	0.052*	0.5
C7	−0.0263 (7)	0.2544 (6)	−0.0659 (4)	0.0376 (12)	
H7B	0.0983	0.2371	−0.0989	0.056*	0.5
H7A	−0.0321	0.1547	−0.0739	0.056*	0.5
H7C	−0.0926	0.3318	−0.1146	0.056*	0.5
H7D	−0.1159	0.2453	−0.0927	0.056*	0.5
H7F	0.0145	0.3277	−0.1177	0.056*	0.5
H7E	0.0750	0.1506	−0.0771	0.056*	0.5
H1	−0.367 (5)	0.427 (4)	0.328 (4)	0.000 (10)*	
H9	0.270 (7)	0.251 (7)	0.412 (5)	0.047 (16)*	

### Di-µ-chlorido-bis[chloridobis(3,4,5-trimethyl-1H-pyrazole-\ κN2)copper(II)] (1) Atomic displacement parameters (Å^2^)

**Table:**

	*U*^11^	*U*^22^	*U*^33^	*U*^12^	*U*^13^	*U*^23^
Cu1	0.0172 (3)	0.0155 (2)	0.0182 (3)	−0.00545 (19)	−0.0067 (2)	−0.00218 (18)
Cl1	0.0258 (5)	0.0207 (5)	0.0177 (5)	−0.0123 (4)	−0.0084 (4)	0.0022 (4)
Cl2	0.0163 (5)	0.0207 (5)	0.0260 (6)	−0.0066 (4)	−0.0051 (4)	−0.0069 (4)
N9	0.0185 (18)	0.0189 (17)	0.027 (2)	−0.0056 (14)	−0.0095 (16)	−0.0028 (14)
N2	0.0162 (17)	0.0203 (17)	0.0198 (18)	−0.0043 (14)	−0.0074 (15)	−0.0010 (14)
N10	0.0198 (18)	0.0211 (17)	0.0218 (19)	−0.0084 (14)	−0.0102 (15)	−0.0005 (14)
N1	0.0163 (19)	0.0218 (18)	0.0173 (19)	−0.0060 (15)	−0.0027 (16)	−0.0018 (14)
C11	0.024 (2)	0.0166 (18)	0.019 (2)	−0.0097 (16)	−0.0095 (17)	0.0002 (15)
C13	0.021 (2)	0.020 (2)	0.024 (2)	−0.0070 (17)	−0.0067 (18)	0.0004 (17)
C3	0.021 (2)	0.022 (2)	0.018 (2)	−0.0072 (17)	−0.0064 (17)	0.0006 (16)
C5	0.024 (2)	0.020 (2)	0.022 (2)	−0.0045 (17)	−0.0115 (18)	−0.0016 (16)
C4	0.027 (2)	0.026 (2)	0.018 (2)	−0.0064 (18)	−0.0089 (19)	−0.0007 (17)
C14	0.031 (2)	0.021 (2)	0.032 (2)	−0.0140 (19)	−0.018 (2)	0.0071 (18)
C12	0.023 (2)	0.0155 (19)	0.021 (2)	−0.0056 (16)	−0.0060 (18)	−0.0032 (16)
C16	0.021 (2)	0.027 (2)	0.042 (3)	−0.0059 (19)	−0.011 (2)	−0.001 (2)
C6	0.022 (2)	0.035 (2)	0.025 (2)	−0.013 (2)	−0.0042 (19)	0.0009 (19)
C8	0.031 (3)	0.035 (3)	0.033 (3)	−0.014 (2)	−0.018 (2)	0.002 (2)
C15	0.032 (3)	0.026 (2)	0.040 (3)	−0.009 (2)	−0.010 (2)	−0.007 (2)
C7	0.040 (3)	0.046 (3)	0.020 (2)	−0.015 (2)	−0.004 (2)	−0.007 (2)

### Di-µ-chlorido-bis[chloridobis(3,4,5-trimethyl-1H-pyrazole-\ κN2)copper(II)] (1) Geometric parameters (Å, º)

**Table:**

Cu1—N10	1.988 (3)	C16—H16B	0.9800
Cu1—N2	2.026 (3)	C16—H16A	0.9800
Cu1—Cl2	2.2939 (12)	C16—H16C	0.9800
Cu1—Cl1	2.3220 (12)	C16—H16D	0.9800
Cu1—Cl1^i^	2.6410 (12)	C16—H16F	0.9800
Cl1—Cu1^i^	2.6410 (12)	C16—H16E	0.9800
N9—C13	1.341 (5)	C6—H6D	0.9800
N9—N10	1.356 (5)	C6—H6F	0.9800
N9—H9	0.86 (6)	C6—H6E	0.9800
N2—C3	1.339 (5)	C6—H6B	0.9800
N2—N1	1.353 (5)	C6—H6A	0.9800
N10—C11	1.341 (5)	C6—H6C	0.9800
N1—C5	1.335 (5)	C8—H8B	0.9800
N1—H1	0.75 (4)	C8—H8A	0.9800
C11—C12	1.403 (6)	C8—H8C	0.9800
C11—C14	1.497 (6)	C8—H8D	0.9800
C13—C12	1.388 (6)	C8—H8F	0.9800
C13—C16	1.492 (6)	C8—H8E	0.9800
C3—C4	1.410 (6)	C15—H15B	0.9800
C3—C6	1.495 (6)	C15—H15A	0.9800
C5—C4	1.383 (6)	C15—H15C	0.9800
C5—C8	1.497 (6)	C15—H15D	0.9800
C4—C7	1.490 (6)	C15—H15F	0.9800
C14—H14D	0.9800	C15—H15E	0.9800
C14—H14F	0.9800	C7—H7B	0.9800
C14—H14E	0.9800	C7—H7A	0.9800
C14—H14B	0.9800	C7—H7C	0.9800
C14—H14A	0.9800	C7—H7D	0.9800
C14—H14C	0.9800	C7—H7F	0.9800
C12—C15	1.503 (6)	C7—H7E	0.9800
			
N10—Cu1—N2	88.98 (14)	C3—C6—H6F	109.5
N10—Cu1—Cl2	161.92 (10)	H6D—C6—H6F	109.5
N2—Cu1—Cl2	88.85 (10)	C3—C6—H6E	109.5
N10—Cu1—Cl1	89.71 (10)	H6D—C6—H6E	109.5
N2—Cu1—Cl1	176.07 (10)	H6F—C6—H6E	109.5
Cl2—Cu1—Cl1	91.26 (4)	C3—C6—H6B	109.5
N10—Cu1—Cl1^i^	100.25 (10)	H6D—C6—H6B	141.1
N2—Cu1—Cl1^i^	99.04 (10)	H6F—C6—H6B	56.3
Cl2—Cu1—Cl1^i^	97.81 (5)	H6E—C6—H6B	56.3
Cl1—Cu1—Cl1^i^	84.83 (4)	C3—C6—H6A	109.5
Cu1—Cl1—Cu1^i^	95.17 (4)	H6D—C6—H6A	56.3
C13—N9—N10	112.5 (3)	H6F—C6—H6A	141.1
C13—N9—H9	130 (4)	H6E—C6—H6A	56.3
N10—N9—H9	118 (4)	H6B—C6—H6A	109.5
C3—N2—N1	105.8 (3)	C3—C6—H6C	109.5
C3—N2—Cu1	133.9 (3)	H6D—C6—H6C	56.3
N1—N2—Cu1	118.3 (3)	H6F—C6—H6C	56.3
C11—N10—N9	105.3 (3)	H6E—C6—H6C	141.1
C11—N10—Cu1	134.0 (3)	H6B—C6—H6C	109.5
N9—N10—Cu1	119.6 (2)	H6A—C6—H6C	109.5
C5—N1—N2	112.0 (4)	C5—C8—H8B	109.5
C5—N1—H1	129 (3)	C5—C8—H8A	109.5
N2—N1—H1	119 (3)	H8B—C8—H8A	109.5
N10—C11—C12	110.4 (3)	C5—C8—H8C	109.5
N10—C11—C14	121.0 (4)	H8B—C8—H8C	109.5
C12—C11—C14	128.6 (4)	H8A—C8—H8C	109.5
N9—C13—C12	106.5 (4)	C5—C8—H8D	109.5
N9—C13—C16	122.2 (4)	H8B—C8—H8D	141.1
C12—C13—C16	131.4 (4)	H8A—C8—H8D	56.3
N2—C3—C4	109.8 (4)	H8C—C8—H8D	56.3
N2—C3—C6	122.3 (4)	C5—C8—H8F	109.5
C4—C3—C6	127.8 (4)	H8B—C8—H8F	56.3
N1—C5—C4	107.2 (4)	H8A—C8—H8F	141.1
N1—C5—C8	121.7 (4)	H8C—C8—H8F	56.3
C4—C5—C8	131.2 (4)	H8D—C8—H8F	109.5
C5—C4—C3	105.2 (4)	C5—C8—H8E	109.5
C5—C4—C7	128.0 (4)	H8B—C8—H8E	56.3
C3—C4—C7	126.8 (4)	H8A—C8—H8E	56.3
C11—C14—H14D	109.5	H8C—C8—H8E	141.1
C11—C14—H14F	109.5	H8D—C8—H8E	109.5
H14D—C14—H14F	109.5	H8F—C8—H8E	109.5
C11—C14—H14E	109.5	C12—C15—H15B	109.5
H14D—C14—H14E	109.5	C12—C15—H15A	109.5
H14F—C14—H14E	109.5	H15B—C15—H15A	109.5
C11—C14—H14B	109.5	C12—C15—H15C	109.5
H14D—C14—H14B	141.1	H15B—C15—H15C	109.5
H14F—C14—H14B	56.3	H15A—C15—H15C	109.5
H14E—C14—H14B	56.3	C12—C15—H15D	109.5
C11—C14—H14A	109.5	H15B—C15—H15D	141.1
H14D—C14—H14A	56.3	H15A—C15—H15D	56.3
H14F—C14—H14A	141.1	H15C—C15—H15D	56.3
H14E—C14—H14A	56.3	C12—C15—H15F	109.5
H14B—C14—H14A	109.5	H15B—C15—H15F	56.3
C11—C14—H14C	109.5	H15A—C15—H15F	141.1
H14D—C14—H14C	56.3	H15C—C15—H15F	56.3
H14F—C14—H14C	56.3	H15D—C15—H15F	109.5
H14E—C14—H14C	141.1	C12—C15—H15E	109.5
H14B—C14—H14C	109.5	H15B—C15—H15E	56.3
H14A—C14—H14C	109.5	H15A—C15—H15E	56.3
C13—C12—C11	105.5 (3)	H15C—C15—H15E	141.1
C13—C12—C15	127.0 (4)	H15D—C15—H15E	109.5
C11—C12—C15	127.6 (4)	H15F—C15—H15E	109.5
C13—C16—H16B	109.5	C4—C7—H7B	109.5
C13—C16—H16A	109.5	C4—C7—H7A	109.5
H16B—C16—H16A	109.5	H7B—C7—H7A	109.5
C13—C16—H16C	109.5	C4—C7—H7C	109.5
H16B—C16—H16C	109.5	H7B—C7—H7C	109.5
H16A—C16—H16C	109.5	H7A—C7—H7C	109.5
C13—C16—H16D	109.5	C4—C7—H7D	109.5
H16B—C16—H16D	141.1	H7B—C7—H7D	141.1
H16A—C16—H16D	56.3	H7A—C7—H7D	56.3
H16C—C16—H16D	56.3	H7C—C7—H7D	56.3
C13—C16—H16F	109.5	C4—C7—H7F	109.5
H16B—C16—H16F	56.3	H7B—C7—H7F	56.3
H16A—C16—H16F	141.1	H7A—C7—H7F	141.1
H16C—C16—H16F	56.3	H7C—C7—H7F	56.3
H16D—C16—H16F	109.5	H7D—C7—H7F	109.5
C13—C16—H16E	109.5	C4—C7—H7E	109.5
H16B—C16—H16E	56.3	H7B—C7—H7E	56.3
H16A—C16—H16E	56.3	H7A—C7—H7E	56.3
H16C—C16—H16E	141.1	H7C—C7—H7E	141.1
H16D—C16—H16E	109.5	H7D—C7—H7E	109.5
H16F—C16—H16E	109.5	H7F—C7—H7E	109.5
C3—C6—H6D	109.5		

Symmetry code: (i) −*x*, −*y*+1, −*z*+1.

### Di-µ-chlorido-bis[chloridobis(3,4,5-trimethyl-1H-pyrazole-\ κN2)copper(II)] (1) Hydrogen-bond geometry (Å, º)

**Table:**

*D*—H···*A*	*D*—H	H···*A*	*D*···*A*	*D*—H···*A*
C14—H14*E*···N2	0.98	2.49	3.199 (5)	129
C14—H14*E*···N1	0.98	2.54	3.401 (6)	147
C14—H14*E*···N2	0.98	2.49	3.199 (5)	129
C14—H14*E*···N1	0.98	2.54	3.401 (6)	147
C16—H16*F*···Cl2^ii^	0.98	2.85	3.776 (5)	158
C6—H6*E*···N9	0.98	2.67	3.521 (6)	146
C6—H6*E*···N10	0.98	2.55	3.240 (6)	128
C6—H6*B*···Cl1^i^	0.98	2.86	3.692 (5)	144
N1—H1···Cl2	0.75 (4)	2.66 (4)	3.102 (4)	120 (3)
N1—H1···Cl2^iii^	0.75 (4)	2.54 (4)	3.214 (4)	151 (3)
N9—H9···Cl1^i^	0.86 (6)	2.94 (6)	3.506 (4)	125 (5)
N9—H9···Cl2^i^	0.86 (6)	2.37 (6)	3.188 (4)	159 (5)

Symmetry codes: (i) −*x*, −*y*+1, −*z*+1; (ii) *x*+1, *y*−1, *z*; (iii) −*x*−1, −*y*+1, −*z*+1.

### Aquatetrakis(3,4,5-trimethyl-1H-pyrazole-κN2)copper(II) dinitrate (2) Crystal data

**Table:**

[Cu(C_6_H_10_N_2_)_4_(H_2_O)](NO_3_)_2_	*F*(000) = 1364
*M**_r_* = 646.21	*D*_x_ = 1.354 Mg m^−^^3^
Monoclinic, *I*2/*a*	Mo *K*α radiation, λ = 0.71073 Å
*a* = 20.107 (7) Å	Cell parameters from 4118 reflections
*b* = 7.8939 (16) Å	θ = 5.6–54.8°
*c* = 20.472 (4) Å	µ = 0.75 mm^−^^1^
β = 102.651 (2)°	*T* = 150 K
*V* = 3170.5 (14) Å^3^	Plate, blue
*Z* = 4	0.16 × 0.10 × 0.03 mm

### Aquatetrakis(3,4,5-trimethyl-1H-pyrazole-κN2)copper(II) dinitrate (2) Data collection

**Table:**

Bruker SMART APEXII CCD diffractometer	3636 independent reflections
Radiation source: fine focus sealed tube	2719 reflections with *I* > 2σ(*I*)
Graphite monochromator	*R*_int_ = 0.057
ω scans	θ_max_ = 27.5°, θ_min_ = 3.2°
Absorption correction: multi-scan (SADABS; Bruker, 2014)	*h* = −26→26
*T*_min_ = 0.654, *T*_max_ = 0.746	*k* = −10→9
13530 measured reflections	*l* = −26→26

### Aquatetrakis(3,4,5-trimethyl-1H-pyrazole-κN2)copper(II) dinitrate (2) Refinement

**Table:**

Refinement on *F*^2^	0 restraints
Least-squares matrix: full	Hydrogen site location: mixed
*R*[*F*^2^ > 2σ(*F*^2^)] = 0.045	H atoms treated by a mixture of independent and constrained refinement
*wR*(*F*^2^) = 0.135	*w* = 1/[σ^2^(*F*_o_^2^) + (0.1*P*)^2^] where *P* = (*F*_o_^2^ + 2*F*_c_^2^)/3
*S* = 0.91	(Δ/σ)_max_ < 0.001
3636 reflections	Δρ_max_ = 0.78 e Å^−^^3^
203 parameters	Δρ_min_ = −0.61 e Å^−^^3^

### Aquatetrakis(3,4,5-trimethyl-1H-pyrazole-κN2)copper(II) dinitrate (2) Special details

**Table:**

Geometry. All esds (except the esd in the dihedral angle between two l.s. planes) are estimated using the full covariance matrix. The cell esds are taken into account individually in the estimation of esds in distances, angles and torsion angles; correlations between esds in cell parameters are only used when they are defined by crystal symmetry. An approximate (isotropic) treatment of cell esds is used for estimating esds involving l.s. planes.
Refinement. Reflections omitted from final refinement on account of beamstop truncation: (h k l) 1 1 0; 2 0 0; 0 1 1; -2 0 2; 0 0 2

### Aquatetrakis(3,4,5-trimethyl-1H-pyrazole-κN2)copper(II) dinitrate (2) Fractional atomic coordinates and isotropic or equivalent isotropic displacement parameters (Å^2^)

**Table:**

	*x*	*y*	*z*	*U*_iso_*/*U*_eq_	Occ. (<1)
Cu1	0.2500	0.50985 (5)	0.5000	0.01754 (15)	
O17	0.2500	0.2345 (3)	0.5000	0.0297 (6)	
N2	0.20222 (9)	0.5683 (3)	0.40597 (10)	0.0207 (4)	
N9	0.11241 (10)	0.6406 (3)	0.50436 (11)	0.0224 (5)	
N10	0.15931 (9)	0.5173 (3)	0.52824 (10)	0.0203 (4)	
N1	0.15514 (11)	0.4676 (3)	0.36661 (12)	0.0234 (5)	
C5	0.13309 (13)	0.5350 (4)	0.30513 (13)	0.0271 (6)	
C11	0.13728 (12)	0.4445 (3)	0.57851 (13)	0.0222 (5)	
C12	0.07613 (12)	0.5194 (3)	0.58642 (14)	0.0241 (5)	
C15	0.03256 (14)	0.4712 (4)	0.63443 (16)	0.0355 (7)	
H15B	0.0540	0.3773	0.6627	0.053*	0.5
H15A	0.0278	0.5688	0.6627	0.053*	0.5
H15C	−0.0126	0.4361	0.6093	0.053*	0.5
H15D	−0.0078	0.5442	0.6271	0.053*	0.5
H15F	0.0184	0.3527	0.6271	0.053*	0.5
H15E	0.0587	0.4854	0.6805	0.053*	0.5
C13	0.06302 (12)	0.6451 (3)	0.53899 (13)	0.0236 (5)	
C3	0.21029 (12)	0.7012 (3)	0.36840 (12)	0.0226 (5)	
C6	0.25867 (14)	0.8425 (4)	0.39353 (15)	0.0321 (6)	
H6D	0.2567	0.9269	0.3580	0.048*	0.5
H6F	0.2460	0.8958	0.4323	0.048*	0.5
H6E	0.3051	0.7975	0.4068	0.048*	0.5
H6B	0.2818	0.8199	0.4400	0.048*	0.5
H6A	0.2925	0.8510	0.3658	0.048*	0.5
H6C	0.2334	0.9493	0.3913	0.048*	0.5
C4	0.16753 (13)	0.6859 (4)	0.30470 (13)	0.0277 (6)	
C14	0.17411 (14)	0.3017 (4)	0.61920 (15)	0.0326 (6)	
H14D	0.1492	0.2678	0.6532	0.049*	0.5
H14F	0.1771	0.2052	0.5899	0.049*	0.5
H14E	0.2201	0.3387	0.6412	0.049*	0.5
H14B	0.2151	0.2733	0.6029	0.049*	0.5
H14A	0.1872	0.3359	0.6663	0.049*	0.5
H14C	0.1442	0.2025	0.6150	0.049*	0.5
C16	0.00746 (13)	0.7757 (4)	0.52520 (16)	0.0336 (6)	
H16B	−0.0235	0.7581	0.5555	0.050*	0.5
H16A	0.0276	0.8891	0.5324	0.050*	0.5
H16C	−0.0180	0.7652	0.4787	0.050*	0.5
H16D	0.0142	0.8501	0.4889	0.050*	0.5
H16F	−0.0369	0.7191	0.5120	0.050*	0.5
H16E	0.0087	0.8430	0.5657	0.050*	0.5
C8	0.08068 (15)	0.4500 (5)	0.25270 (15)	0.0398 (7)	
H8B	0.0717	0.5188	0.2119	0.060*	0.5
H8A	0.0973	0.3381	0.2430	0.060*	0.5
H8C	0.0385	0.4369	0.2688	0.060*	0.5
H8D	0.0666	0.3438	0.2705	0.060*	0.5
H8F	0.0410	0.5245	0.2394	0.060*	0.5
H8E	0.0999	0.4257	0.2136	0.060*	0.5
C7	0.15739 (18)	0.8152 (5)	0.24974 (16)	0.0456 (8)	
H7B	0.1890	0.9098	0.2633	0.068*	0.5
H7A	0.1662	0.7632	0.2090	0.068*	0.5
H7C	0.1104	0.8572	0.2411	0.068*	0.5
H7D	0.1214	0.7770	0.2123	0.068*	0.5
H7F	0.1442	0.9236	0.2666	0.068*	0.5
H7E	0.2000	0.8296	0.2345	0.068*	0.5
O20	0.10612 (12)	0.1364 (3)	0.37019 (11)	0.0430 (6)	
O21	0.07101 (11)	−0.1135 (3)	0.38872 (12)	0.0459 (6)	
O19	0.14070 (10)	0.0169 (3)	0.46693 (12)	0.0411 (5)	
N18	0.10557 (11)	0.0134 (3)	0.40797 (13)	0.0297 (5)	
H1	0.1460 (14)	0.383 (4)	0.3781 (14)	0.021 (8)*	
H9	0.1192 (14)	0.702 (4)	0.4685 (15)	0.031 (8)*	
H17	0.2176 (14)	0.176 (4)	0.4879 (15)	0.029 (8)*	

### Aquatetrakis(3,4,5-trimethyl-1H-pyrazole-κN2)copper(II) dinitrate (2) Atomic displacement parameters (Å^2^)

**Table:**

	*U*^11^	*U*^22^	*U*^33^	*U*^12^	*U*^13^	*U*^23^
Cu1	0.0159 (2)	0.0155 (2)	0.0221 (2)	0.000	0.00595 (15)	0.000
O17	0.0234 (13)	0.0158 (13)	0.0498 (18)	0.000	0.0077 (13)	0.000
N2	0.0208 (10)	0.0173 (10)	0.0246 (11)	−0.0015 (8)	0.0062 (9)	0.0010 (8)
N9	0.0201 (10)	0.0177 (11)	0.0305 (12)	0.0032 (8)	0.0081 (9)	0.0019 (9)
N10	0.0155 (9)	0.0210 (11)	0.0251 (11)	0.0038 (7)	0.0059 (8)	0.0048 (8)
N1	0.0221 (10)	0.0208 (12)	0.0266 (12)	−0.0041 (8)	0.0039 (9)	0.0003 (9)
C5	0.0236 (12)	0.0353 (16)	0.0228 (13)	0.0038 (11)	0.0059 (10)	−0.0024 (11)
C11	0.0203 (11)	0.0198 (12)	0.0275 (13)	−0.0017 (9)	0.0074 (10)	0.0007 (10)
C12	0.0184 (11)	0.0267 (14)	0.0286 (13)	−0.0056 (9)	0.0079 (10)	−0.0052 (10)
C15	0.0283 (13)	0.0450 (19)	0.0368 (16)	−0.0099 (12)	0.0153 (12)	−0.0027 (13)
C13	0.0180 (11)	0.0220 (13)	0.0314 (14)	−0.0015 (9)	0.0068 (10)	−0.0065 (10)
C3	0.0252 (11)	0.0197 (13)	0.0254 (13)	0.0040 (10)	0.0110 (10)	0.0028 (10)
C6	0.0364 (14)	0.0246 (15)	0.0361 (16)	−0.0030 (11)	0.0098 (12)	0.0016 (11)
C4	0.0297 (13)	0.0279 (14)	0.0267 (14)	0.0079 (11)	0.0092 (11)	0.0060 (11)
C14	0.0322 (13)	0.0293 (15)	0.0384 (16)	0.0059 (12)	0.0123 (12)	0.0120 (12)
C16	0.0260 (12)	0.0285 (16)	0.0478 (17)	0.0066 (11)	0.0112 (12)	−0.0046 (13)
C8	0.0327 (15)	0.054 (2)	0.0302 (16)	−0.0032 (14)	0.0023 (13)	−0.0073 (14)
C7	0.0562 (19)	0.046 (2)	0.0343 (17)	0.0078 (16)	0.0099 (15)	0.0147 (15)
O20	0.0552 (13)	0.0259 (11)	0.0454 (13)	−0.0093 (9)	0.0053 (11)	0.0081 (9)
O21	0.0524 (13)	0.0278 (12)	0.0618 (15)	−0.0154 (10)	0.0218 (12)	−0.0101 (10)
O19	0.0271 (10)	0.0432 (13)	0.0493 (13)	−0.0067 (8)	0.0003 (9)	0.0158 (10)
N18	0.0257 (11)	0.0198 (12)	0.0458 (14)	0.0016 (9)	0.0128 (10)	0.0018 (10)

### Aquatetrakis(3,4,5-trimethyl-1H-pyrazole-κN2)copper(II) dinitrate (2) Geometric parameters (Å, º)

**Table:**

Cu1—N2	2.008 (2)	C6—H6B	0.9800
Cu1—N2^i^	2.008 (2)	C6—H6A	0.9800
Cu1—N10	2.031 (2)	C6—H6C	0.9800
Cu1—N10^i^	2.031 (2)	C4—C7	1.499 (4)
Cu1—O17	2.174 (3)	C14—H14D	0.9800
O17—H17	0.79 (3)	C14—H14F	0.9800
N2—C3	1.332 (3)	C14—H14E	0.9800
N2—N1	1.358 (3)	C14—H14B	0.9800
N9—C13	1.341 (3)	C14—H14A	0.9800
N9—N10	1.369 (3)	C14—H14C	0.9800
N9—H9	0.92 (3)	C16—H16B	0.9800
N10—C11	1.336 (3)	C16—H16A	0.9800
N1—C5	1.349 (4)	C16—H16C	0.9800
N1—H1	0.75 (3)	C16—H16D	0.9800
C5—C4	1.379 (4)	C16—H16F	0.9800
C5—C8	1.489 (4)	C16—H16E	0.9800
C11—C12	1.405 (4)	C8—H8B	0.9800
C11—C14	1.497 (4)	C8—H8A	0.9800
C12—C13	1.373 (4)	C8—H8C	0.9800
C12—C15	1.502 (4)	C8—H8D	0.9800
C15—H15B	0.9800	C8—H8F	0.9800
C15—H15A	0.9800	C8—H8E	0.9800
C15—H15C	0.9800	C7—H7B	0.9800
C15—H15D	0.9800	C7—H7A	0.9800
C15—H15F	0.9800	C7—H7C	0.9800
C15—H15E	0.9800	C7—H7D	0.9800
C13—C16	1.501 (3)	C7—H7F	0.9800
C3—C4	1.401 (4)	C7—H7E	0.9800
C3—C6	1.495 (4)	O20—N18	1.243 (3)
C6—H6D	0.9800	O21—N18	1.234 (3)
C6—H6F	0.9800	O19—N18	1.258 (3)
C6—H6E	0.9800		
			
N2—Cu1—N2^i^	153.41 (13)	H14D—C14—H14F	109.5
N2—Cu1—N10	89.73 (8)	C11—C14—H14E	109.5
N2^i^—Cu1—N10	89.51 (8)	H14D—C14—H14E	109.5
N2—Cu1—N10^i^	89.50 (8)	H14F—C14—H14E	109.5
N2^i^—Cu1—N10^i^	89.73 (8)	C11—C14—H14B	109.5
N10—Cu1—N10^i^	176.69 (12)	H14D—C14—H14B	141.1
N2—Cu1—O17	103.30 (6)	H14F—C14—H14B	56.3
N2^i^—Cu1—O17	103.29 (6)	H14E—C14—H14B	56.3
N10—Cu1—O17	91.66 (6)	C11—C14—H14A	109.5
N10^i^—Cu1—O17	91.66 (6)	H14D—C14—H14A	56.3
Cu1—O17—H17	126 (2)	H14F—C14—H14A	141.1
C3—N2—N1	106.0 (2)	H14E—C14—H14A	56.3
C3—N2—Cu1	129.93 (17)	H14B—C14—H14A	109.5
N1—N2—Cu1	124.01 (17)	C11—C14—H14C	109.5
C13—N9—N10	111.2 (2)	H14D—C14—H14C	56.3
C13—N9—H9	131.9 (18)	H14F—C14—H14C	56.3
N10—N9—H9	116.9 (18)	H14E—C14—H14C	141.1
C11—N10—N9	105.21 (19)	H14B—C14—H14C	109.5
C11—N10—Cu1	132.58 (16)	H14A—C14—H14C	109.5
N9—N10—Cu1	120.67 (16)	C13—C16—H16B	109.5
C5—N1—N2	111.5 (2)	C13—C16—H16A	109.5
C5—N1—H1	126 (2)	H16B—C16—H16A	109.5
N2—N1—H1	122 (2)	C13—C16—H16C	109.5
N1—C5—C4	106.5 (2)	H16B—C16—H16C	109.5
N1—C5—C8	122.1 (3)	H16A—C16—H16C	109.5
C4—C5—C8	131.4 (3)	C13—C16—H16D	109.5
N10—C11—C12	110.7 (2)	H16B—C16—H16D	141.1
N10—C11—C14	123.0 (2)	H16A—C16—H16D	56.3
C12—C11—C14	126.2 (2)	H16C—C16—H16D	56.3
C13—C12—C11	105.1 (2)	C13—C16—H16F	109.5
C13—C12—C15	126.6 (2)	H16B—C16—H16F	56.3
C11—C12—C15	128.3 (3)	H16A—C16—H16F	141.1
C12—C15—H15B	109.5	H16C—C16—H16F	56.3
C12—C15—H15A	109.5	H16D—C16—H16F	109.5
H15B—C15—H15A	109.5	C13—C16—H16E	109.5
C12—C15—H15C	109.5	H16B—C16—H16E	56.3
H15B—C15—H15C	109.5	H16A—C16—H16E	56.3
H15A—C15—H15C	109.5	H16C—C16—H16E	141.1
C12—C15—H15D	109.5	H16D—C16—H16E	109.5
H15B—C15—H15D	141.1	H16F—C16—H16E	109.5
H15A—C15—H15D	56.3	C5—C8—H8B	109.5
H15C—C15—H15D	56.3	C5—C8—H8A	109.5
C12—C15—H15F	109.5	H8B—C8—H8A	109.5
H15B—C15—H15F	56.3	C5—C8—H8C	109.5
H15A—C15—H15F	141.1	H8B—C8—H8C	109.5
H15C—C15—H15F	56.3	H8A—C8—H8C	109.5
H15D—C15—H15F	109.5	C5—C8—H8D	109.5
C12—C15—H15E	109.5	H8B—C8—H8D	141.1
H15B—C15—H15E	56.3	H8A—C8—H8D	56.3
H15A—C15—H15E	56.3	H8C—C8—H8D	56.3
H15C—C15—H15E	141.1	C5—C8—H8F	109.5
H15D—C15—H15E	109.5	H8B—C8—H8F	56.3
H15F—C15—H15E	109.5	H8A—C8—H8F	141.1
N9—C13—C12	107.7 (2)	H8C—C8—H8F	56.3
N9—C13—C16	121.7 (2)	H8D—C8—H8F	109.5
C12—C13—C16	130.5 (2)	C5—C8—H8E	109.5
N2—C3—C4	110.0 (2)	H8B—C8—H8E	56.3
N2—C3—C6	122.8 (2)	H8A—C8—H8E	56.3
C4—C3—C6	127.2 (2)	H8C—C8—H8E	141.1
C3—C6—H6D	109.5	H8D—C8—H8E	109.5
C3—C6—H6F	109.5	H8F—C8—H8E	109.5
H6D—C6—H6F	109.5	C4—C7—H7B	109.5
C3—C6—H6E	109.5	C4—C7—H7A	109.5
H6D—C6—H6E	109.5	H7B—C7—H7A	109.5
H6F—C6—H6E	109.5	C4—C7—H7C	109.5
C3—C6—H6B	109.5	H7B—C7—H7C	109.5
H6D—C6—H6B	141.1	H7A—C7—H7C	109.5
H6F—C6—H6B	56.3	C4—C7—H7D	109.5
H6E—C6—H6B	56.3	H7B—C7—H7D	141.1
C3—C6—H6A	109.5	H7A—C7—H7D	56.3
H6D—C6—H6A	56.3	H7C—C7—H7D	56.3
H6F—C6—H6A	141.1	C4—C7—H7F	109.5
H6E—C6—H6A	56.3	H7B—C7—H7F	56.3
H6B—C6—H6A	109.5	H7A—C7—H7F	141.1
C3—C6—H6C	109.5	H7C—C7—H7F	56.3
H6D—C6—H6C	56.3	H7D—C7—H7F	109.5
H6F—C6—H6C	56.3	C4—C7—H7E	109.5
H6E—C6—H6C	141.1	H7B—C7—H7E	56.3
H6B—C6—H6C	109.5	H7A—C7—H7E	56.3
H6A—C6—H6C	109.5	H7C—C7—H7E	141.1
C5—C4—C3	106.0 (2)	H7D—C7—H7E	109.5
C5—C4—C7	127.4 (3)	H7F—C7—H7E	109.5
C3—C4—C7	126.5 (3)	O21—N18—O20	121.1 (3)
C11—C14—H14D	109.5	O21—N18—O19	118.8 (2)
C11—C14—H14F	109.5	O20—N18—O19	120.0 (2)

Symmetry code: (i) −*x*+1/2, *y*, −*z*+1.

### Aquatetrakis(3,4,5-trimethyl-1H-pyrazole-κN2)copper(II) dinitrate (2) Hydrogen-bond geometry (Å, º)

**Table:**

*D*—H···*A*	*D*—H	H···*A*	*D*···*A*	*D*—H···*A*
C6—H6*E*···N9^i^	0.98	2.50	3.355 (4)	145
C6—H6*E*···N10^i^	0.98	2.60	3.274 (4)	126
C6—H6*F*···O19^ii^	0.98	2.56	3.367 (4)	140
C6—H6*E*···N9^i^	0.98	2.50	3.355 (4)	145
C6—H6*E*···N10^i^	0.98	2.60	3.274 (4)	126
C14—H14*B*···O17	0.98	2.38	3.193 (3)	140
C16—H16*B*···O20^iii^	0.98	2.62	3.525 (4)	153
C16—H16*B*···N18^iii^	0.98	2.66	3.342 (4)	127
C16—H16*D*···O21^ii^	0.98	2.57	3.430 (4)	146
C8—H8*D*···O20	0.98	2.60	3.411 (4)	140
N1—H1···O20	0.75 (3)	2.10 (3)	2.801 (3)	158 (3)
N9—H9···N2	0.92 (3)	2.54 (3)	3.039 (3)	114 (2)
N9—H9···O21^ii^	0.92 (3)	2.24 (3)	3.033 (3)	144 (2)
N9—H9···O19^ii^	0.92 (3)	2.52 (3)	3.150 (3)	126 (2)
O17—H17···O19	0.79 (3)	1.97 (3)	2.755 (3)	174 (3)
C6—H6*F*···O19^ii^	0.98	2.56	3.367 (4)	140
C6—H6*E*···N9^i^	0.98	2.50	3.355 (4)	145
C6—H6*E*···N10^i^	0.98	2.60	3.274 (4)	126
C6—H6*E*···N9^i^	0.98	2.50	3.355 (4)	145
C6—H6*E*···N10^i^	0.98	2.60	3.274 (4)	126
C14—H14*B*···O17	0.98	2.38	3.193 (3)	140
C16—H16*B*···O20^iii^	0.98	2.62	3.525 (4)	153
C16—H16*B*···N18^iii^	0.98	2.66	3.342 (4)	127
C16—H16*D*···O21^ii^	0.98	2.57	3.430 (4)	146
C8—H8*D*···O20	0.98	2.60	3.411 (4)	140
N1—H1···O20	0.75 (3)	2.10 (3)	2.801 (3)	158 (3)
N9—H9···N2	0.92 (3)	2.54 (3)	3.039 (3)	114 (2)
N9—H9···O21^ii^	0.92 (3)	2.24 (3)	3.033 (3)	144 (2)
N9—H9···O19^ii^	0.92 (3)	2.52 (3)	3.150 (3)	126 (2)
O17—H17···O19	0.79 (3)	1.97 (3)	2.755 (3)	174 (3)

Symmetry codes: (i) −*x*+1/2, *y*, −*z*+1; (ii) *x*, *y*+1, *z*; (iii) −*x*, −*y*+1, −*z*+1.
